# Enhanced peripheral levels of BDNF and proBDNF: elucidating neurotrophin dynamics in cocaine use disorder

**DOI:** 10.1038/s41380-023-02367-7

**Published:** 2024-01-04

**Authors:** Mauro Pettorruso, Andrea Miuli, Katia Clemente, Gianluca Mancusi, Giuseppe Migliara, Francesco Di Carlo, Giulia Pernaci, Teresa Di Crosta, Mario Santorelli, Giacomo d’Andrea, Luisa De Risio, Mariaceleste Ciavarella, Valentina Baccolini, Ilenia Di Meo, Ivana Cataldo, Stefano L. Sensi, Giovanni Martinotti

**Affiliations:** 1 Department of Neurosciences, Imaging and Clinical Sciences, University “G. D’Annunzio” of Chieti - Pescara, Chieti, Italy; 2 Department of Mental Health, ASL 2 Abruzzo Lanciano-Vasto-Chieti, Chieti, Italy; 3 Unit of Clinical Pathology, ASL 2 Abruzzo Lanciano-Vasto-Chieti, Chieti, Italy; 4 Department of Public Health and Infectious Diseases, Sapienza University of Rome, Rome, Italy; 5 School of Medicine and Surgery, Psychiatric Residency Training Program, University of Milano Bicocca, Monza, 20900 Italy; 6 Department of Mental Health and Addiction, ASL Roma 5, Rome, Italy; 7 Psychopharmacology, Drug Misuse and Novel Psychoactive Substances Research Unit, School of Life and Medical Sciences, University of Hertfordshire, Hatfield, AL10 9AB UK

**Keywords:** Neuroscience, Biotechnology, Biomarkers

## Abstract

Brain-derived neurotrophic factor (BDNF) and its precursor, proBDNF, are known to significantly contribute to brain homeostasis, neuroplasticity, and neuronal remodeling. Although these neurotrophins are thought to have opposing roles, both play a critical part in shaping long-lasting behavioral changes following substance use. In this context, our study sought to explore the implications of these neurotrophins in the pathophysiology of cocaine use disorder (CUD). We conducted a case-control study, which included 28 individuals seeking treatment for CUD and 38 matched healthy participants. We measured peripheral neurotrophin concentrations via an enzyme-linked immunosorbent assay. Additionally, all participants were screened for cocaine-associated pathways (e.g., cocaine intake, craving intensity), along with associated psychopathological data. Our findings highlighted an increased concentration of BDNF and proBDNF in CUD individuals when compared to healthy controls (BDNF: 18092.80 ± 6844.62 vs. 11334.42 ± 5061.85 pg/ml, *p* < 0.001; proBDNF: 87.03 ± 33.23 vs. 55.70 ± 23.26 ng/ml, *p* < 0.001). We further corroborated the relationship between neurotrophin levels and CUD using a linear regression model. Nevertheless, there was no significant difference in the proBDNF to BDNF ratio between the two groups. Interestingly, our study also demonstrated the influence of factors like usage of psychotropic medications, history of psychiatric hospitalizations, and psychiatric diagnoses on neurotrophin dynamics. In conclusion, our study underscores the significance of neurotrophin fluctuations in CUD. The observed increase in BDNF and proBDNF levels could play a pivotal role in driving craving and relapse risk. Thus, a nuanced understanding of these neurobiological underpinnings in CUD might contribute to the development of more targeted and effective therapeutic strategies.

## Introduction

Cocaine use disorder (CUD) substantially contributes to global morbidity and disability [1]. Despite its detrimental effects, cocaine consumption triggers structural and functional neurobiological changes that consolidate craving and substance-seeking behaviors. The immediate cocaine-related effects are primarily linked to positive reinforcement mechanisms, while its prolonged use better aligns with the emergence of withdrawal-induced negative emotionality or ingrained behavioral habits. These elements constitute the “addiction cycle” (i.e., binge intoxication, withdrawal, anticipation/craving) [2], in which each stage has been linked to specific alterations in neurotrophin action [3, 4].

Neurotrophins, as a family of growth factors, orchestrate cellular activities such as proliferation, differentiation, survival, repair, and programmed cell death in neuronal and non-neuronal cells. By regulating neuroplasticity, these factors induce lasting behavioral adaptations following substance use and are instrumental in mediating the dynamics of pursuit and reward behaviors, as well as in adjusting homeostatic imbalances.

Brain-derived neurotrophic factor (BDNF), the most prevalent neurotrophin in the brain, facilitates cell growth and differentiation, synaptic connectivity, and neuroplasticity and modulates neurotransmission, neuronal repair, and neurodegenerative processes [5]. Its crucial function in facilitating recovery from depressive states has recently been elucidated [6]. BDNF activates several intracellular pathways (e.g., MAP kinase, PI3-kinase, PLC-γ) by binding to its receptor TrkB on pre-synaptic and post-synaptic membranes [7]. It is synthesized from its precursor proBDNF, produced by neurons, and stored in pre-synaptic secretory vesicles. ProBDNF primarily serves as a reserve for the mature molecule, but it also has an independent biological effect via its interaction with the p75NTR receptor [8]. By binding p75NTR, it contributes to brain plasticity and neuronal remodeling, activating apoptosis and the elimination of damaged, malfunctioning, or ineffective synapses [9]. BDNF and proBDNF have distinct release patterns in the circulatory system. Mature BDNF, stored in neuronal and platelet α-granules, is released also after platelet activation. However, proBDNF, constituting only 10% of the total circulating concentration, does not correlate with platelet activation. Studies, including Le Blanc et al. (2020), indicate that proBDNF’s presence in the bloodstream is not a result of platelet activation and that its intracellular concentration remains stable post cellular triggering [10, 11]. Hence, proBDNF might primarily indicate neuronal rather than platelet activation.

Despite the assumption that proBDNF and BDNF have opposing roles [12], both are deemed essential for preserving functional homeostasis within the central nervous system.

Notably, BDNF plays a pivotal role in learning and memory processes, both of which are crucial in the pathophysiology of addiction. With this understanding, BDNF function has been comprehensively investigated in a wide array of addictive disorders, including CUD [13]. Research has yielded mixed results regarding BDNF levels in CUD patients compared to healthy controls. Some studies report lower BDNF levels in individuals with CUD [14, 15], whereas others indicate higher levels [16]. These seemingly contradictory findings may be understood in the context of different stages of the “addiction cycle” [4]. BDNF levels significantly drop soon after cocaine use [3, 14, 15], persist at low levels throughout the intoxication phase, and rebound during withdrawal [15, 17].

The impact of various factors on peripheral neurotrophin levels has been extensively documented. These factors, encompassing age, sex, physical activity, tobacco usage, and sleep quality, have demonstrated modulatory effects on the transcription, secretion, and circulating levels of BDNF and proBDNF [18–21]. Research into diverse substance addictions has unveiled significant heterogeneity in neurotrophic alterations, which are largely influenced by the substance type, consumption pattern, and stage of addiction. Notably, a consistent trend of declining serum BDNF levels has been linked to the progression of chronic substance use [3].

Lastly, beyond the absolute levels of BDNF and proBDNF, contemporary hypotheses emphasize the potential significance of the proBDNF/BDNF ratio. The equilibrium of neurotrophin levels undergoes substantial shifts during growth and in reaction to physiological and pathological states, thereby guiding neuroplasticity toward either advantageous or detrimental neuromodulatory pathways [12]. In the postnatal developmental phase, the production of proBDNF exceeds that of the mature isoform, while this ratio is reversed during adolescence and adulthood [22]. Intriguingly, this proBDNF/BDNF ratio inversion aligns with the progression of adolescence, a period with an inherently elevated risk for the onset of mental illness. Consistently, the proBDNF/BDNF ratio has been proposed as a biomarker able to distinguish conditions such as bipolar disorder and major depression [23], whereas no research has explored its role in CUD.

Building upon the existing understanding and growing evidence surrounding the role of mature BDNF and the current gap in knowledge regarding the involvement of its precursor proBDNF in the pathophysiology of CUD, we herein present the results of a case-control study. In this work, we assessed neurotrophin levels (i.e., BDNF and proBDNF) in individuals with CUD and matched healthy controls. Additionally, we performed a proof-of-concept analysis to investigate the relationship between neurotrophin levels and other clinical dimensions, including psychopathological features.

## Materials and methods

### Participants and procedures

In this case-control study, 28 treatment-seeking individuals diagnosed with CUD and 38 healthy controls were recruited on a voluntary basis in April and May 2023 at the Psychiatric Clinic in “SS Annunziata” Hospital, Chieti, Italy. The CUD participants were recruited in this ancillary study from a wider sample of treatment-seeking patients participating in the Brainswitch project, a double-blind, sham-controlled trial testing the ability of repetitive transcranial magnetic stimulation to treat CUD (preliminary data published in Martinotti et al. [24]). Sample size was calculated assuming a level of BDNF of 26 ± 9 ng in healthy controls and of 36 ± 9 ng in CUDs [16], an alpha error of 5% and a power of 80%, resulting in a sample size of 18 (9 per group). However, given the ancillary nature of the present study, we enrolled all the 28 CUDs who volunteered given the availability of data.

The inclusion criteria for the participants with CUD were: (i) age between 18 and 65 years; (ii) diagnosis of moderate to severe CUD according to DSM-5 criteria; (iii) abstinence from cocaine for at least 48 h; (iv) no current use of pro-convulsive drugs; (v) no history of seizures; and (vi) for female patients, no pregnancy/lactation. Due to the limited sample size, the CUD group was stratified for age, sex, and smoking habit, and a stratified recruitment strategy was applied for the healthy controls. For the healthy control group, the inclusion criteria were: (i) age between 18 and 65 years; (ii) absence of psychiatric, neurological, or other medical diagnoses; (iii) no history of substance use; (iv) no psychopharmaceutical intake; and (v) for female patients, no pregnancy/lactation.

Before being enrolled, all participants underwent a detailed anamnestic interview. For the healthy controls, the absence of any psychiatric diagnosis and substance misuse were assessed by a trained psychiatrist and verified using the Patient Health Questionnaire-9 (PHQ-9).

### Measures

(a) Sociodemographic and psychometric assessment

The anamnestic form included sociodemographic information (i.e., age, employment, and family status), psychiatric history (i.e., presence of a psychiatric diagnosis, current psychopharmacological treatment, and cigarette smoking), and clinical features of CUD. The psychometric assessment included the Fagerström Test for Nicotine Dependence and the PHQ-9. The Fagerström test is a standard instrument for assessing the intensity of physical addiction to nicotine. It contains six items evaluating compulsion to use, dependence, and quantity of cigarette consumption. The items are summed to a total of 0–10, with 10 the maximum possible value for the patient’s physical dependence to nicotine [25]. The PHQ-9 is a nine-item questionnaire designed to screen for depression. The standard cut-off for screening for major depression is 10 or above [26].

(b) Enzyme-linked immunosorbent assay

ProBDNF and BDNF were evaluated in venous blood samples. After centrifugation at 1000×g for 15 min, the serum levels of proBDNF and BDNF were determined using enzyme-linked immunosorbent assay (ELISA) performed on 96-well plates pre-coated with highly specific monoclonal antibodies. The BDNF-ELISA kit was coated with specific murine monoclonal anti-BDNF antibodies, whereas the human proBDNF-ELISA kit was coated with anti-proBDNF antibodies. Horseradish peroxidase (HRP) conjugated to streptavidin was added to the solution, which binds to the solid phase via the biotin-streptavidin bond. The samples were then incubated, and the unbound conjugates were washed off. Finally, the substrate 3,3′,5,5′-tetramethylbenzydine (TMB) was added to the solution, which undergoes an enzymatic reaction catalyzed by HRP from which a blue color is produced that turns yellow after the addition of an acidic stop solution. The signal strength was measured on a microplate reader at 450 nm and was quantitatively proportional to the amount of BDNF or proBDNF captured in the well. BDNF values were quantified in relation to a standard curve calibrated with a known amount of protein. The detection limit was 2 pg/mL. Measurements were performed in duplicate and are expressed in pg/mL (BDNF) and ng/mL (proBDNF). ProBDNF values were quantified in relation to a standard curve calibrated with a known amount of protein. The detection limit was 0.094 ng/mL. No cross-reactivity or significant interference was observed among BDNF, proBDNF, and other neurotrophins.

### Statistical analysis

Descriptive statistics were calculated using mean and standard deviation (SD) or median and interquartile range (IQR) for continuous variables, as appropriate for the normality of the distribution, and using proportions for dichotomous and categorical variables. The BDNF/proBDNF ratio was expressed using a scaling factor of 1000 to improve data legibility. The normality of continuous variables was verified using the Shapiro-Wilk test. Univariable analysis was performed using the Student’s *t* test or the Wilcoxon rank-sum test, as appropriate, to compare continuous variables between the CUD and control groups, whereas Pearson’s chi-square test or Fisher’s exact test was used for dichotomous and categorical variables, as appropriate. Three multivariable linear regression models were built to estimate the adjusted beta coefficients (aβ) and associated 95% confidence intervals (CIs) of factors influencing the values of BDNF (Model 1), proBDNF (Model 2), and the BDNF/proBDNF ratio (Model 3). To account for the stratified recruitment, cluster-adjusted standard errors were estimated. Variables were included in the model based on expert opinion. For all final models, the variables included were the following: cocaine consumption (0 = no; 1 = yes), age (continuous), sex (0 = female; 1 = male), presence of male offspring (0 = no, 1 = yes), civil status (0 = single, 1 = married, 2 = divorced), employment (0 = no, 1 = yes), psychopharmaceutical intake (0 = no, 1 = yes), previous psychiatric hospitalization (0 = no, 1 = yes), previous psychiatric diagnosis (0 = no, 1 = yes), and smoking habit (0 = no, 1 = yes). BDNF (continuous) was included only in the model for the BDNF/proBDNF ratio.

All analyses were performed using STATA 17.0 (StataCorp LLC, College Station, TX, USA). A two-sided *p*-value < 0.05 was considered statistically significant.

## Results

### Sociodemographic and clinical characteristics

The recruited sample comprised 66 participants (Table 1). The CUD group included 28 patients (males/females, 27/1; mean age, 37.9 ± 7.9 years), while the control group included 38 participants (males/females, 34/4; mean age, 39.5 ± 11.5 years). The groups were homogenous in terms of all sociodemographic characteristics analyzed. Specifically, we did not detect any significant difference in relation to gender (*p* = 0.385) or mean age (*p* = 0.519), having sons (*n* = 15, 57.69% vs. *n* = 13, 37.14%; *p* = 0.110), marital status (single: *n* = 13, 50.00% vs. *n* = 22, 62.86%; married: *n* = 12, 46.15% vs. *n* = 12, 34.29%; divorced: *n* = 1, 3.85% vs. *n* = 1, 2.86%; *p* = 0.604) and employment (*n* = 20, 80.00% vs. *n* = 28, 80.00%; *p* = 1.00). The CUD group showed a higher frequency of psychopharmaceutical intake (*n* = 9, 34.62% vs. *n* = 0, 0.00%; *p* < 0.001), previous psychiatric hospitalization (*n* = 4, 15.38% vs. *n* = 0, 0.00%; *p* = 0.013), and previous psychiatric diagnosis (*n* = 11, 40.74% vs. *n* = 0, 0.00%; *p* < 0.001), but a comparable proportion of smokers (*n* = 17, 60.71% vs. *n* = 20, 52.63%; *p* = 0.513).

**Table 1:** Comparison between patients with cocaine use disorder and healthy controls.

	Cocaine consumption *N* (%)		*P*-value
	Yes	No	
	28	38	
**Gender**			0.385^a^
Female	1 (3.57%)	4 (10.53%)	
Male	27 (96.43%)	34 (89.47%)	
**Age, years**			
Mean (SD)	37.93 (7.89)	39.47 (11.49)	0.519^b^
**Having sons (*N*** **=** 61)			0.110^c^
No	11 (42.31%)	22 (62.86%)	
Yes	15 (57.69%)	13 (37.14%)	
**Marital Status (*N*** **=** 61)			0.604^a^
Single	13 (50.00%)	22 (62.86%)	
Married	12 (46.15%)	12 (34.29%)	
Divorced	1 (3.85%)	1 (2.86%)	
**Employment (*N*** **=** 60)			1.000^c^
No	5 (20.00%)	7 (20.00%)	
Yes	20 (80.00%)	28 (80.00%)	
**Psychopharmaceuticals intake (*N*** **=** 64)			<0.001^a^
No	17 (65.38%)	38 (100.00%)	
Yes	9 (34.62%)	0 (0.00%)	
**Previous psychiatric hospitalizations (*N*** **=** 64)			0.013^a^
No	22 (84.62%)	38 (100.00%)	
Yes	4 (15.38%)	0 (0.00%)	
**Previous psychiatric diagnoses (*N*** **=** 65)			<0.001^a^
No	16 (59.26%)	38 (100.00%)	
Yes	11 (40.74%)	0 (0.00%)	
**Smoking habit**			0.513^c^
No	11 (39.29%)	18 (47.37%)	
Yes	17 (60.71%)	20 (52.63%)	
**BDNF levels (pg/ml)**			<0.001^d^
Mean (SD)	18092.80 (6844.62)	11334.42 (5061.85)	
**ProBDNF levels (ng/ml)**			<0.001^b^
Mean (SD)	87.03 (33.23)	55.70 (23.26)	
**ProBDNF/BDNF Ratio per mille**			0.559^e^
Median (IQR)	4.74	5.04	
(4.40, 5.15)		(4.12, 6.69)	

*BDNF* brain-derived neurotrophic factor, *IQR* interquartile range, *SD* standard deviation.

^a^Fisher’s exact test.

^b^Student’s *t* test, unequal variance.

^c^Pearson’s chi-squared test.

^d^Student’s *t* test, equal variance.

^e^Mann–Whitney *U* test.

### Neurotrophin levels

**Fig. 1:**
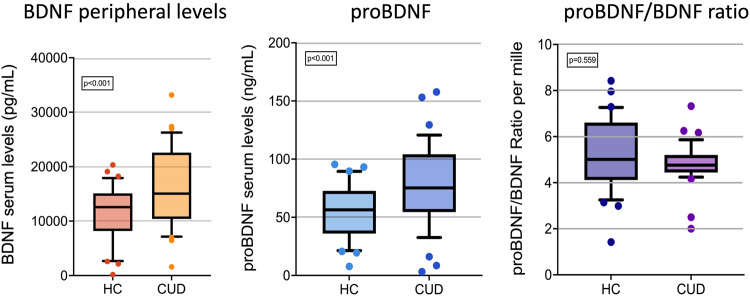
Peripheral BDNF and proBDNF levels in CUD and HC subjects.

Significantly higher mean values of both BDNF (18092.80 ± 6844.62 vs. 11334.42 ± 5061.85) and proBDNF (87.03 ± 33.23 vs. 55.70 ± 23.26) were found in the CUD group (*p* < 0.001; Table 1, Fig. 1). In contrast, the median BDNF/proBDNF ratio did not differ significantly between the groups (4.74, interquartile range:4.40–5.15 vs. 5.04, interquartile range:4.12–6.69; *p* = 0.559) (Table 1, Fig. 1). The linear regression models confirmed the associations of BDNF and proBDNF levels with CUD presence (Table 2).

**Table 2:** Linear regression models predicting neurotrophin levels and proBNDF/BDNF ratio.

	BDNF levels^a^			ProBDNF levels^a^			ProBNDF/BDNF Ratio^b^		
	aβ	95% CI	*P*-value	aβ	95% CI	*P*-value	aβ	95% CI	*P*-value
Cocaine consumption (yes)	4.26	0.39–8.46	**0.033**	23.59	3.69–43.49	**0.023**	2.35	−5.18 to 9.88	0.519
Age (years, continuous)	0.18	0.03–0.33	**0.023**	0.21	−0.53 to 0.95	0.557	−0.02	−0.28 to 0.24	0.864
Gender (female vs. male)	3.05	1.36–4.76	**0.002**	9.06	−4.02 to 22.14	0.162	−25.06	−48.24 to −1.88	**0.036**
Having sons (yes)	2.29	0.03–4.56	**0.047**	3.69	−15.66 to 23.05	0.692	−2.44	−9.61 to 4.74	0.483
Civil status (ref: single)									
Married	−1.92	−5.26 to 1.41	0.242	−2.08	−27.65 to 23.49	0.866	2.90	−16.44 to 8.08	0.482
Divorced	1.44	−5.30 to 2.41	0.441	0.08	−33.86 to 34.01	0.996	−4.18	−4.66 to 7.72	0.608
Employment (yes)	−1.76	−6.15 to 2.64	0.411	1.50	−16.17 to 19.16	0.860	1.53	−3.08 to 8.88	0.320
Psychopharmaceuticals intake (yes)	8.78	5.22–12.33	**<0.001**	42.71	19.95–65.46	**0.001**	7.64	−8.21 to 23.49	0.324
Previous psychiatric hospitalizations (yes)	11.78	4.96–18.60	**0.002**	60.64	27.01–94.26	**0.001**	−0.70	−7.00 to 5.60	0.817
Previous psychiatric diagnoses (yes)	−7.56	−13.43 to −1.68	**0.015**	−43.43	−66.76 to −20.10	**0.001**	−7.34	−22.06 to 7.39	0.308
Smoking habit (yes)	−1.82	−4.67 to 1.03	0.195	−8.29	−25.21 to 8.70	0.318	2.77	−2.52 to 8.06	0.285
BDNF levels (μg/ml, continuous)	/	/	/	/	/	/	−0.56	−1.82 to 0.71	0.366

*aβ* adjusted regression coefficients, *CI* confidence interval, *BDNF* brain-derived neurotrophic factor.

^a^Expressed as ng/ml.

^b^Expressed as per 1000.

Significant values (*p* < 0.05) shown in bold.

Specifically, higher BDNF levels were associated with cocaine use (aβ = 4.26, 95% CI: 0.39–8.46, *p* = 0.033), older age (aβ = 0.18, 95% CI: 0.03–0.33, *p* = 0.023), female sex (aβ = 3.05, 95% CI: 1.36–4.76, *p* = 0.002), presence of male offspring (aβ = 2.29, 95% CI: 0.03–4.56, *p* = 0.047), psychopharmaceutical intake (aβ = 8.78, 95% CI: 5.22–12.33, *p* < 0.001), and previous psychiatric hospitalization (aβ = 11.78, 95% CI: 4.96–18.60, *p* = 0.002) (Model 1). On the other hand, a previous psychiatric diagnosis was negatively associated with mean BDNF levels (aβ = −7.56, 95%CI: −13.43 to –1.68, *p* < 0.015). No association was found for marital status (married vs. single, *p* = 0.242; and divorced vs. single; *p* = 0.441), employment (*p* = 0.411) and smoking habit (*p* = 0.195).

In Model 2, higher ProBDNF levels were associated with cocaine use (aβ = 23.59, 95% CI: 3.69–43.49, *p* = 0.023), psychopharmaceutical intake (aβ = 42.71, 95% CI: 19.95–65.46, *p* = 0.001), and previous psychiatric hospitalization (aβ = 60.64, 95% CI: 27.01–94.26, *p* = 0.001), whereas a previous psychiatric diagnosis was negatively associated with the outcome (aβ = −43.43, 95% CI: −66.76 to −20.10, *p* = 0.001). Older age (*p* = 0.557), gender (*p* = 0.162), having sons (*p* = 0.692), marital status (married vs. single, *p* = 0.866; and divorced vs. single; *p* = 0.996), employment (*p* = 0.860) and smoking habit (*p* = 0.318) did not seem to influence the ProBDNF levels.

Lastly, sex (female: aβ = −25.06, 95% CI: −48.24 to −1.88, *p* = 0.036) was the only predictor associated with BDNF/proBDNF ratio (Model 3). No association was found for cocaine consumption (*p* = 0.519), older age (*p* = 0.864), having sons (*p* = 0.483), marital status (married vs. single, *p* = 0.482; and divorced vs. single; *p* = 0.608), employment (*p* = 0.320), psychopharmaceuticals intake (*p* = 0.324), previous psychiatric hospitalization (*p* = 0.817), previous psychiatric disorder diagnosis (*p* = 0.308), smoking habit (*p* = 0.285) and higher BDNF levels (*p* = 0.366).

## Discussion

The main finding in the present study was an increase in peripheral levels of both proBDNF and BDNF in CUD patients who were abstinent from cocaine for a minimum of 2 days before testing compared with healthy individuals. Given the balance between the central production of BDNF and proBDNF and their circulating levels [11], the brain production of these neurotrophins can be postulated to increase during cocaine withdrawal. This observation aligns with a previous study that reported that CUD patients who were abstinent for 3 weeks had high blood BDNF concentrations [16]. Interestingly, individuals who exhibited higher peripheral neurotrophin levels were more likely to relapse in the short term.

To our knowledge, the present study was the first to report the concurrent upregulation of proBDNF and BDNF in CUD individuals, with no impact on the proBDNF/BDNF ratio. Fluctuations of BDNF levels are observed under a wide range of physiological and pathological conditions. These fluctuations are associated with neuroprotection, positive neuromodulation, neural growth, and various neurological and psychiatric conditions that are characterized by degenerative and neurotoxic pathways [27–29]. Conversely, the increase of both BDNF and proBDNF during withdrawal may represent a sign of a non-neurodegenerative process, putatively ascribed to the activation of stress-response systems (e.g., the extended amygdala). The upregulation of BDNF and proBDNF could be attributed to both an increase in genetic transcription and more efficient proBDNF maturation [30]. This allostatic equilibrium does not appear to alter the proBDNF/BDNF ratio, which has been associated with processes that counteract cocaine-induced neurotoxic damage [31].

The main finding in the present study was an increase in both proBDNF and BDNF in peripheral blood during abstinence in human CUD subjects. Recent research identified close associations between peripheral BDNF levels and CUD, although the precise dynamic mechanisms of these changes across the addiction cycle remain largely unexplored. Several preclinical and clinical studies reported fluctuations of BDNF levels across various phases of cocaine addiction [3, 14, 16]. For example, BDNF has been implicated in craving and seeking-related behaviors in preclinical studies, with periods of cocaine abstinence that are characterized by high BDNF levels during craving episodes. Consequently, BDNF peaks correspond to greater relapse risk, and BDNF injections in specific brain regions in mice can modulate cocaine-seeking behaviors [32, 33]. Furthermore, proBDNF could serve as a “reservoir” for the mature molecule, and an increase in proBDNF suggests a larger BDNF deposit that is likely associated with greater genetic transcription (e.g., epigenetic regulation). We previously proposed that BDNF could be a phase-specific marker of cocaine abstinence and a potential indicator of relapse [4, 16]. Supporting this possibility, blood levels of BDNF significantly declined during chronic cocaine exposure but tended to increase during early abstinence [14, 15]. BDNF and proBDNF appear to be linked to craving and relapse risk in early withdrawal periods, but the persistence of higher levels over several months reveals a deeper and more complex disruption of neurotrophin production, reflecting enduring and long-term reactive structural and functional changes in neurotrophic equilibrium. Several hypotheses may explain these findings. From a translational perspective, BDNF has been shown to be a pivotal modulator of dependence-related behaviors. Infusions of BDNF in the ventral tegmental area in mice induces or amplifies craving and cocaine-seeking behaviors [34, 35], whereas BDNF injections in the medial prefrontal cortex inhibit cocaine-seeking behaviors [32]. Therefore, BDNF may foster cocaine dependence-related behaviors, particularly behaviors that are related to craving and the risk of relapse. BDNF levels are typically lower during chronic cocaine use, and initiation of the withdrawal phase reduces BDNF levels back to healthy control values [36], thereby reestablishing allostatic balance that is induced by CUD. Consequently, the state of abstinence could disrupt this equilibrium, and an increase in BDNF, concomitant with greater craving, could trigger a behavioral mechanism that seeks to reestablish this balance.

Additionally, BDNF increases could represent a pathophysiological response to neurotoxic effects of cocaine. Psychostimulants have been reported to induce long-term extracellular and intracellular neurochemical damage primarily via glutamate-mediated pathways in dopaminergic reward systems, thereby modifying neuronal viability and neuroplasticity [31]. Chronic exposure to cocaine has been linked to oxidative damage pathways that are directly associated with neuroinflammation and neurodegeneration [37]. Therefore, an increase in BDNF during abstinence could activate positive neuromodulatory mechanisms as an adaptive response to excitotoxic neuronal damage. Furthermore, clinical manifestations of withdrawal, such as dysphoria and irritability, mirror neurobiological adaptive mechanisms that involve greater stress sensitivity and hyperactivation of the hypothalamic-pituitary-adrenal axis, which could account for the increase in BDNF levels [38, 39].

Our findings preliminarily support the potential of the proBDNF/BDNF ratio as a phase-specific marker of neuroplasticity in CUD [4]. The simultaneous detection of BDNF and proBDNF variations could provide more valuable insights than their separate values [23, 40]. BDNF has significant positive neuromodulatory properties (e.g., long-term potentiation, sprouting, and neural survivability). proBDNF plays a contrasting role by inducing negative neuroplasticity mechanisms (e.g., long-term depression, pruning, and neural apoptosis) through its specific molecular pathways [30]. The balance of these two factors has been recognized as pivotal in terms of neuroplasticity. When the proBDNF/BDNF ratio skews toward proBDNF, a predominance of negative neuroplasticity/apoptosis is observed (i.e., cognitive and memory performance deficits) [41]. A ratio that favors BDNF could signal positive neuroplasticity/neuroprotection (i.e., during adolescence and young adulthood) [22].

Interestingly, our results underscore that psychiatric ward admissions are independently associated with high neurotrophin levels, supporting a significant association between the use of psychotropic medications and the upregulation of BDNF and proBDNF in CUD. Antidepressants, lithium, and antipsychotics have been shown to exert neuromodulatory effects on BDNF levels [42–44]. Our findings, in turn, suggest that these effects are similarly manifested in patients with CUD. Notably, elevations of BDNF are linked to greater craving and a higher risk of relapse [16], which should be carefully considered when interventions are selected that target these systems.

The positive neuromodulation and synaptic trophism that are induced by BDNF could offer ways to enhance the neurobiological resilience of the brain that is impacted by addiction. However, these same pathways might also intensify craving during initial withdrawal phases, potentially accelerating relapse. Selective serotonin reuptake inhibitors, which enhance BDNF levels, could potentially amplify cocaine craving and thereby facilitate relapse. Consistent with these observations, potential strategies to “stabilize” neurotrophins should be explored, with the goal of normalizing, rather than amplifying, BDNF and proBDNF levels [4]. Such approaches as transcranial magnetic stimulation, which locally modulates neuronal excitability without ubiquitously increasing BDNF levels [45, 46], may represent more targeted therapeutics for anhedonic/depressive comorbid CUD conditions [24, 47–49] and could better align with the complex physiology of brain neurotrophins.

Finally, the regression model that was used herein identified psychiatric diagnosis as a negative influencer of BDNF and proBDNF levels during abstinence, highlighting the intricate, yet still elusive, relationship between neurotrophin balance and psychiatric illness. Research indicates low circulating BDNF levels in different mental health conditions [50–54]. When these disorders co-occur with CUD, referred to as dual diagnosis, we observe distinct neurotrophin fluctuations, emphasizing the impact of psychiatric disease on central BDNF production. Notably, although BDNF levels are elevated in dual diagnosis patients during abstinence, they follow a different pattern and are lower than in non-comorbid CUD patients. These findings call for a comprehensive psychiatric evaluation of cocaine users when neurotrophin levels are being assessed.

### Limitations

The present study has limitations that must be acknowledged when interpreting the findings. Although the sample was adequately powered, its size was relatively small. Therefore, our findings should be corroborated in larger, well-controlled cohorts. The presence of BDNF in the bloodstream can be influenced by various variables that are difficult to control, such as smoking [19], the hypothalamic-pituitary-adrenal axis and stress hormones [55], ovarian cycles [56], and exercise [21]. BDNF is primarily stored in platelets and other cellular components [11], whose quantities may fluctuate based on different blood conditions [21]. Conversely, proBDNF is predominantly found in the central nervous system, and only a minimal portion enters the bloodstream, suggesting that it could represent a more stable and less variable marker of central BDNF production. Additionally, our results may predominantly reflect specific characteristics of treatment-seeking individuals with CUD as opposed to non-treatment-seeking individuals. Thus, generalization to all individuals with CUD should be done with caution. However, one strength of our study was the recruitment of participants from a naturalistic, non-preselected setting. Another limitation was the absence of metrics that assessed the precise duration of abstinence. The inclusion criterion was a minimum of 48 h since the last cocaine intake, but this could result in wide variance in the intensity of withdrawal symptoms among participants. Moreover, although the extant literature suggests no link between plasma proBDNF concentrations and platelet activation, the detection of proBDNF dosage in experimental settings remains inconsistent. We also cannot rule out its rapid conversion into the mature form following release. Lastly, although our study design aligned with the baseline evaluation phase of a clinical trial, we did not conduct multiple assessments of neurotrophins. Future research would benefit from the inclusion of more extensive and longitudinal measurements to adequately characterize neurotrophin fluctuations over time.

## Data Availability

Data are available on request from the authors at sequent email addresses: Mauro Pettorruso, MD, PhD; Department of Neurosciences, Imaging and Clinical Sciences, University “G. D’Annunzio” of Chieti-Pescara, Chieti, Italy mauro.pettorruso@unich.it (first author)
